# Supplementary material for: A Humanized Yeast Phenomic Model of Deoxycytidine Kinase to Predict Genetic Buffering of Nucleoside Analog Cytotoxicity
Source: Genes (Basel). 2019 Sep 30;10(10):770. doi: 10.3390/genes10100770 (PMC6826991; doi:10.3390/genes10100770)
Supplement: Supplementary file 1 [file genes-10-00770-s001.zip › Additional_Files/Additional_File7_GOTermHeatmaps.zip!/Additional_File7_GOTermHeatmaps/DNA-directed_5'-3'_RNA_polymerase_activity.pdf]

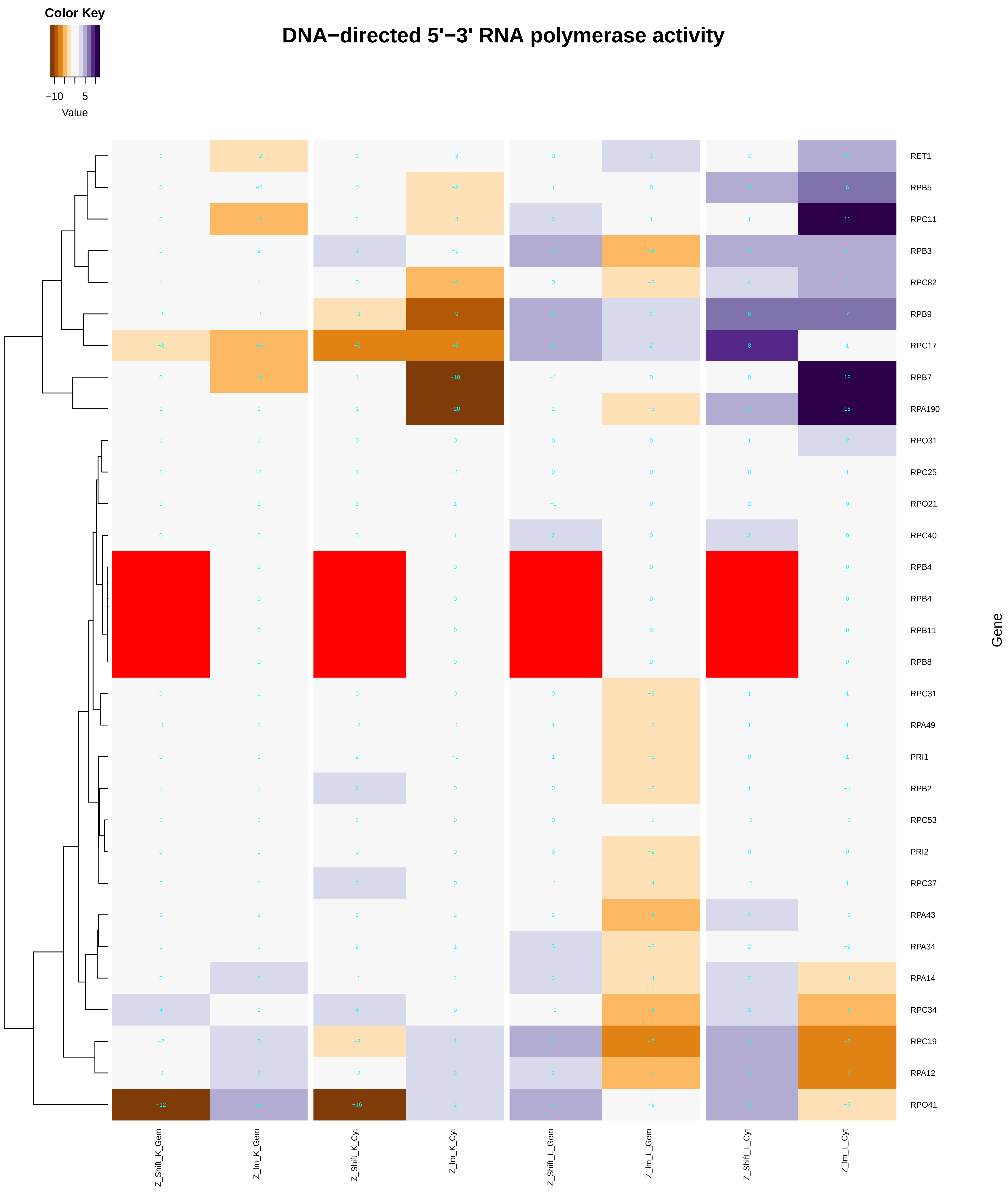

Color Key
−10
5
Value
DNA−directed 5'−3' RNA polymerase activity
RET1
RPB5
RPC11
RPB3
RPC82
RPB9
RPC17
RPB7
RPA190
RPO31
RPC25
RPO21
RPC40
RPB4
RPB4
RPB11
RPB8
RPC31
RPA49
PRI1
RPB2
RPC53
PRI2
RPC37
RPA43
RPA34
RPA14
RPC34
RPC19
RPA12
RPO41
Gene
Z_Shift_K_Gem
Z_Im_K_Gem
Z_Shift_K_Cyt
Z_Im_K_Cyt
Z_Shift_L_Gem
Z_Im_L_Gem
Z_Shift_L_Cyt
Z_Im_L_Cyt

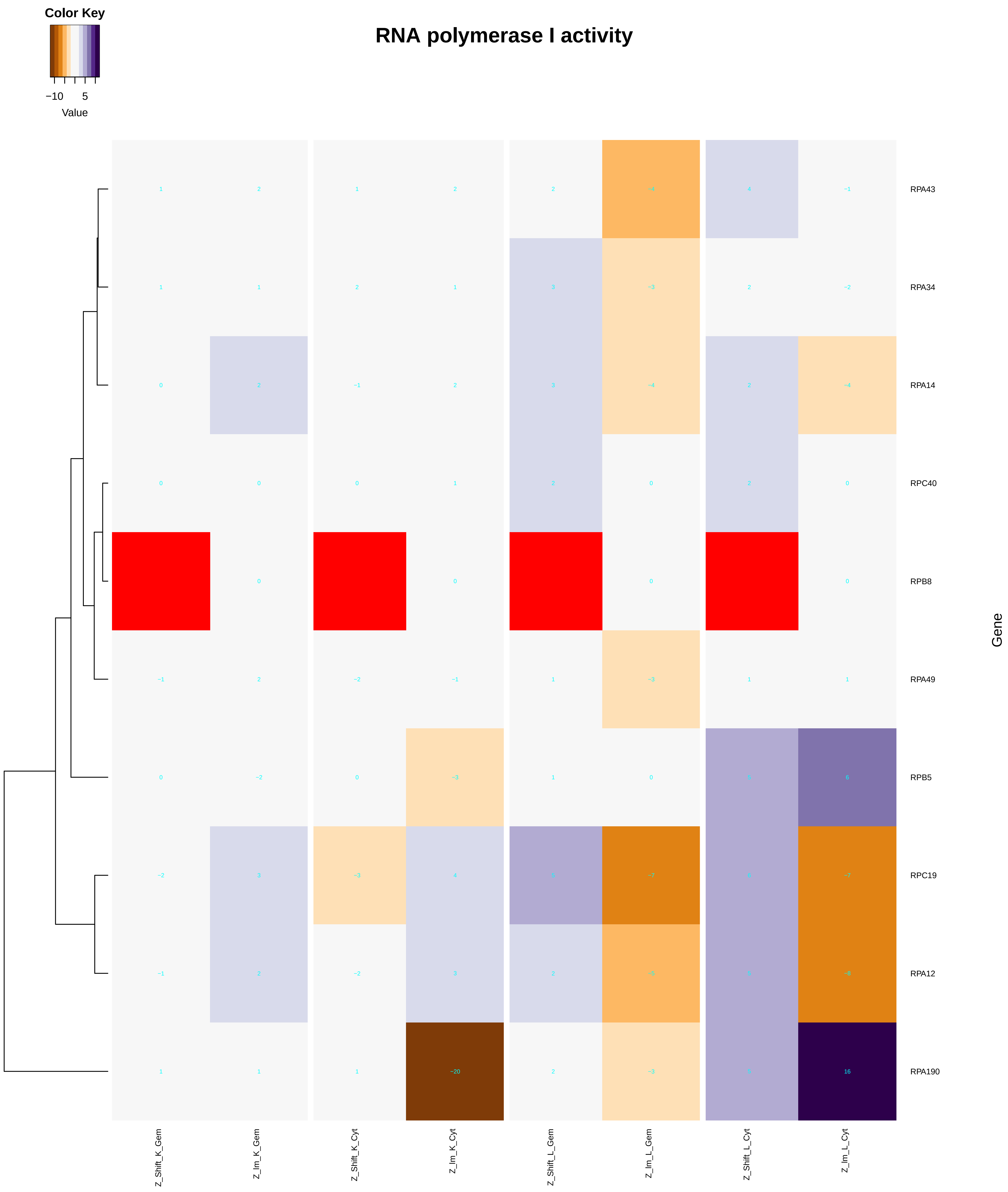
Color Key
−10
5
Value
RNA polymerase I activity
RPA43
RPA34
RPA14
RPC40
RPB8
RPA49
RPB5
RPC19
RPA12
RPA190
Gene
Z_Shift_K_Gem
Z_Im_K_Gem
Z_Shift_K_Cyt
Z_Im_K_Cyt
Z_Shift_L_Gem
Z_Im_L_Gem
Z_Shift_L_Cyt
Z_Im_L_Cyt

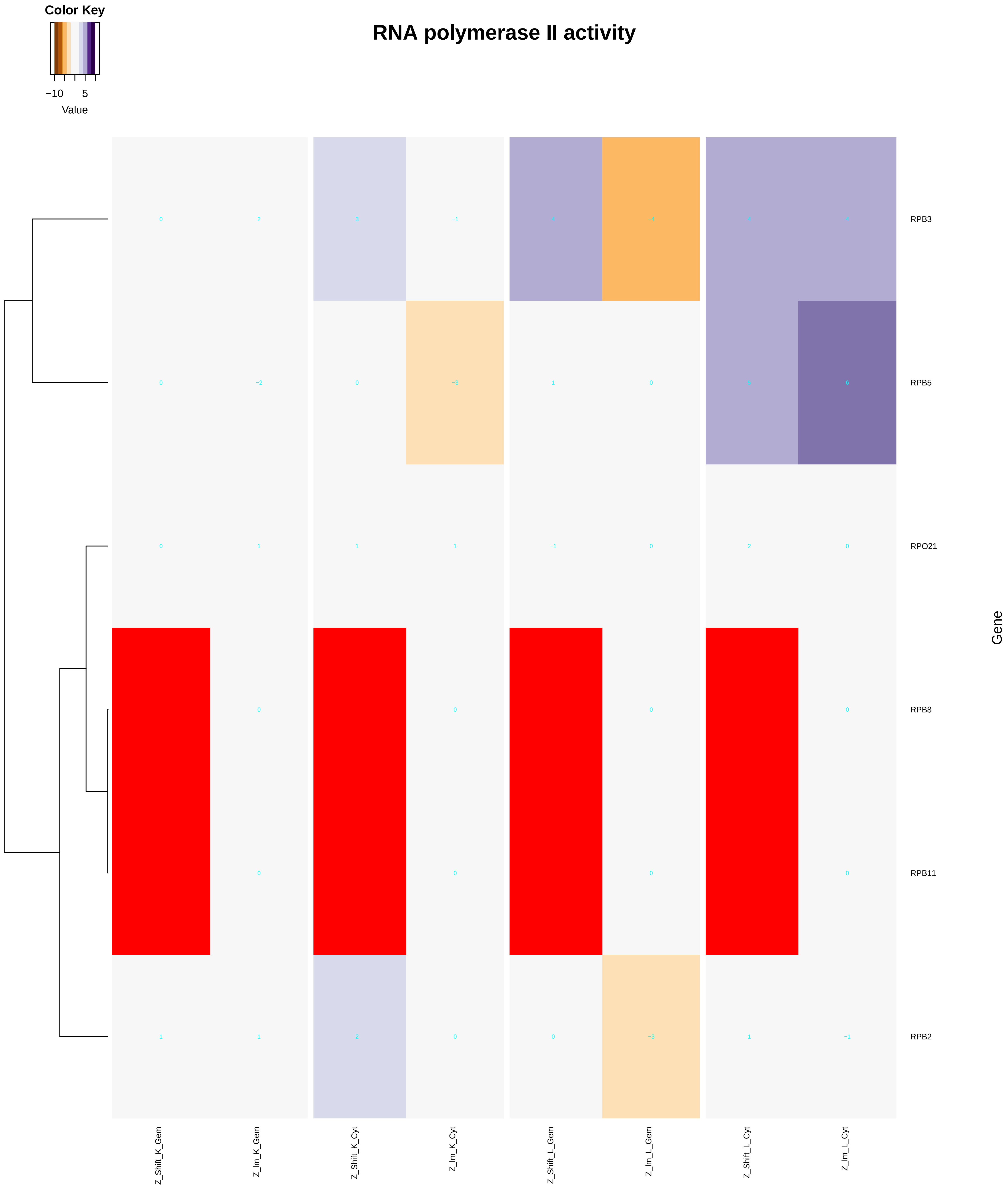
Color Key
−10
5
Value
RNA polymerase II activity
RPB3
RPB5
RPO21
RPB8
RPB11
RPB2
Gene
Z_Shift_K_Gem
Z_Im_K_Gem
Z_Shift_K_Cyt
Z_Im_K_Cyt
Z_Shift_L_Gem
Z_Im_L_Gem
Z_Shift_L_Cyt
Z_Im_L_Cyt

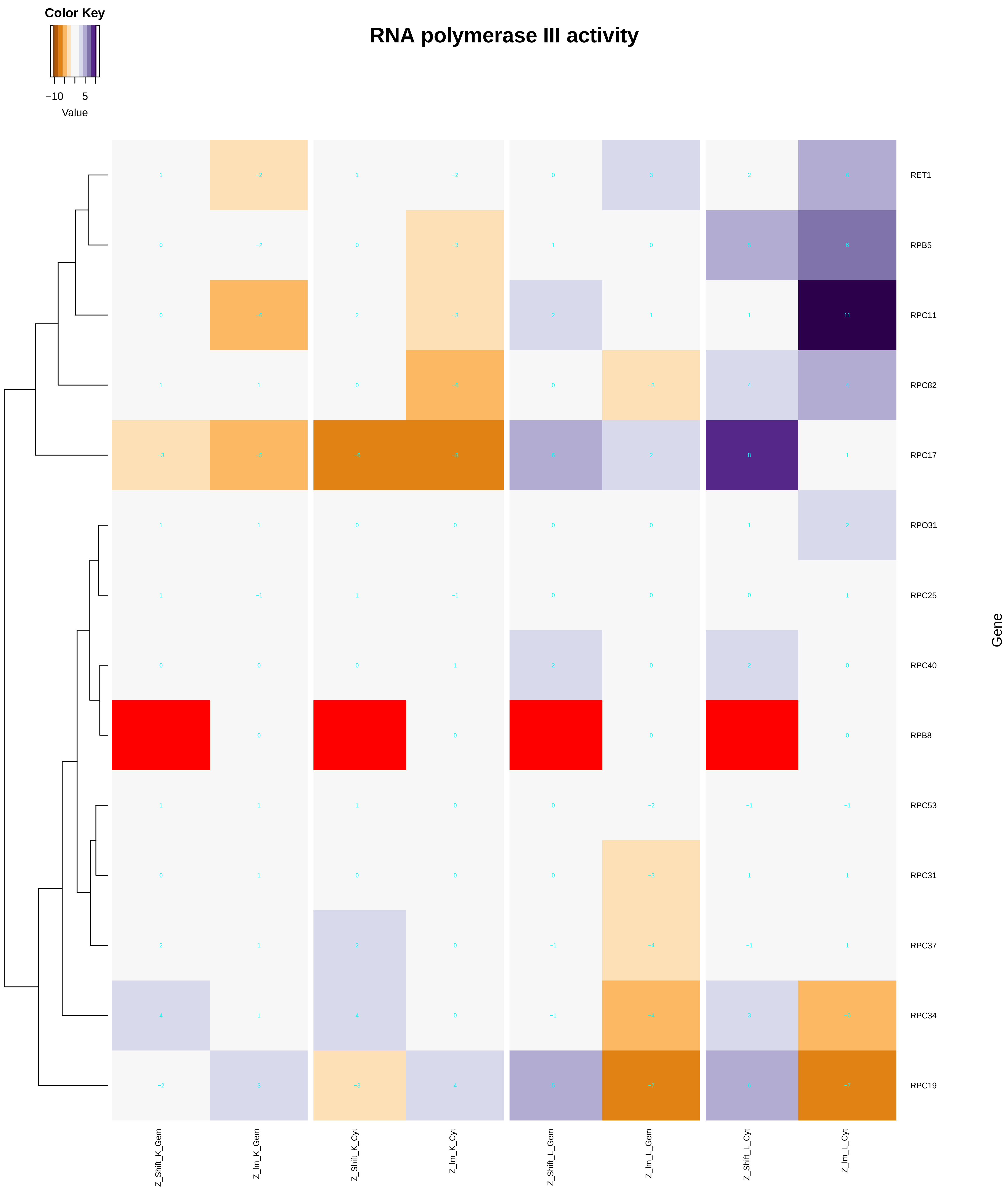

RNA polymerase III activity
Color Key
−10
5
Value
RET1
RPB5
RPC11
RPC82
RPC17
RPO31
RPC25
RPC40
RPB8
RPC53
RPC31
RPC37
RPC34
RPC19
Gene
Z_Shift_K_Gem
Z_Im_K_Gem
Z_Shift_K_Cyt
Z_Im_K_Cyt
Z_Shift_L_Gem
Z_Im_L_Gem
Z_Shift_L_Cyt
Z_Im_L_Cyt

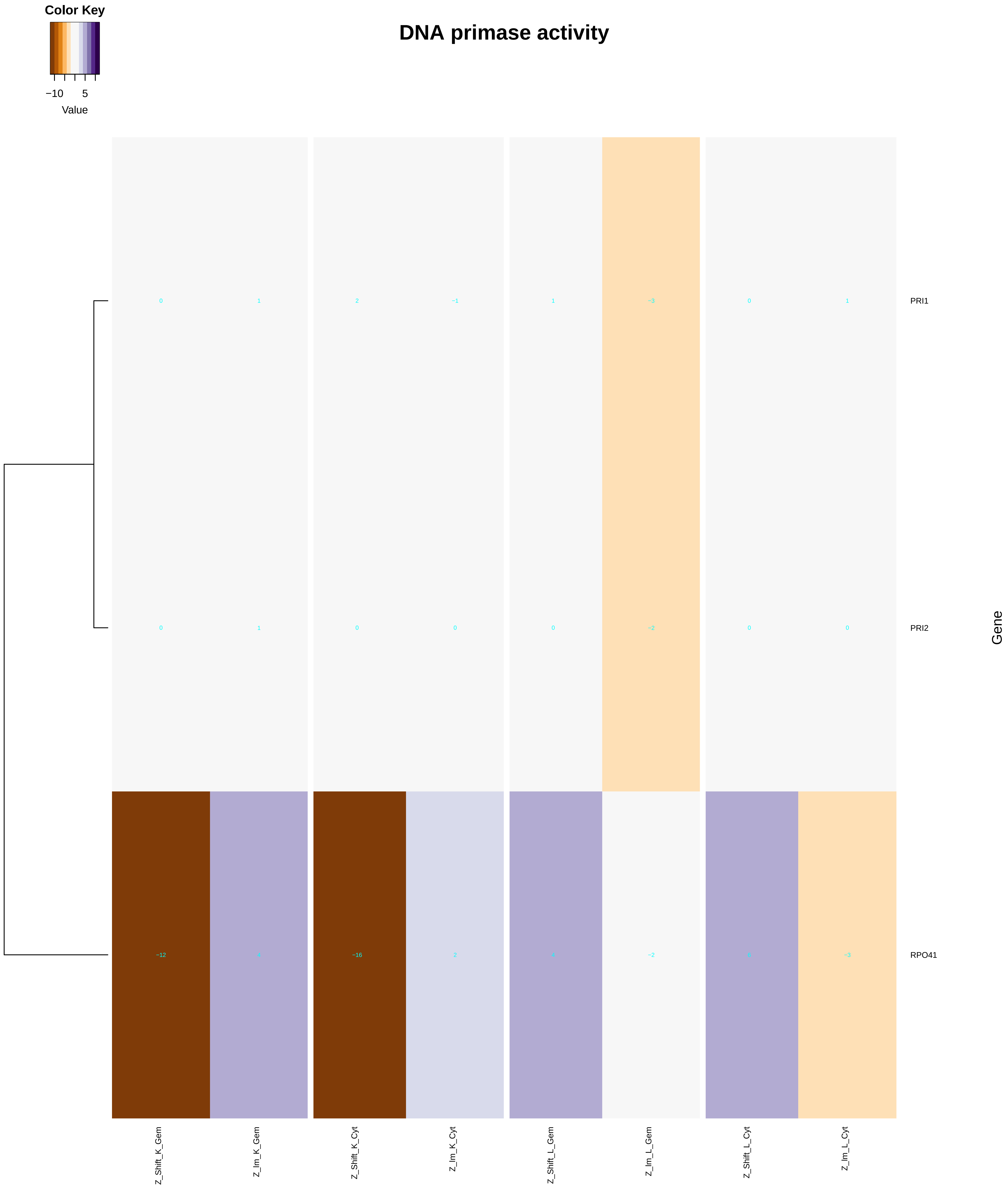
Color Key
−10
5
Value
DNA primase activity
0
1
2
−1
1
−3
0
1
PRI1
0
1
0
0
0
−2
0
0
PRI2
−12
4
−16
2
4
−2
6
−3
RPO41
Gene
Z_Shift_K_Gem
Z_Im_K_Gem
Z_Shift_K_Cyt
Z_Im_K_Cyt
Z_Shift_L_Gem
Z_Im_L_Gem
Z_Shift_L_Cyt
Z_Im_L_Cyt
